# Knowledge, attitude and practice in tuberculosis treatment among HIV-positive patients: Association with sputum conversion-A mixed-methods study

**DOI:** 10.6026/973206300222324

**Published:** 2026-04-30

**Authors:** Loisen Nathan Daniel, Amirtha Manoharan, Anant Fulse, Mohamed Reda Khafagy, Dhivya Mohan, Aswathy T Menon

**Affiliations:** 1 Department of Emergency Medicine, Best Hospital, Tamil Nadu, India; 2 Department of Medicine, Sree Balaji Medical College and Hospital, Chromepet, Chennai, Tamil Nadu, India; 3 Department of Anatomy, NKP Salve Institute of Medical Sciences & RC and Lata Mangeshkar Hospital, Nagpur, India; 4 Department of Emergency Medicine, Essex, United Kingdom; 5 Department of Medicine, Aarupadai Veedu Medical College and Hospital, Tamil Nadu, India; 6 Department of General Medicine, Sree Balaji Medical College, Chennai, Tamil Nadu, India

**Keywords:** Tuberculosis (TB), human immunodeficiency virus (HIV), awareness, compliance, sputum conversion, knowledge, attitudes and practice (KAP)

## Abstract

Tuberculosis remains a major cause of morbidity and mortality among individuals living with HIV. Adherence to anti-tuberculosis therapy is essential for successful treatment and sputum conversion. Therefore, it is of interest to assess knowledge, attitudes and practices (KAP) regarding tuberculosis treatment among 120 HIV-positive patients receiving first-line therapy and examined their association with sputum conversion. Structured questionnaires and follow-up interviews were administered. Sputum microscopy was performed at baseline, 2 months and 6 months. The mean total KAP score was 17.3 ± 3.8. Sputum conversion occurred in 78% at 2 months and 92% at 6 months. Higher KAP scores were significantly associated with earlier sputum conversion (p < 0.05). Thus, we show that patient awareness and adherence behaviours are strongly associated with treatment outcomes. Structured counselling and adherence-focused interventions may improve tuberculosis control in HIV-infected populations.

## Background:

Tuberculosis remains a major global health challenge, particularly among individuals living with HIV. HIV infection increases susceptibility to tuberculosis through immune suppression and accelerates disease progression [1]. Co-infected patients experience higher rates of treatment complications, relapse and mortality [2]. Effective tuberculosis control requires early diagnosis, strict adherence to therapy and sustained follow-up [3]. Standard treatment under the Directly Observed Treatment, Short-course (DOTS) strategy has improved outcomes. However, non-adherence remains a major obstacle. Incomplete treatment increases the risk of drug resistance, treatment failure and ongoing transmission [4]. Patient knowledge plays a central role in adherence. Understanding symptoms, treatment duration and potential side effects improves compliance [5]. Misconceptions regarding tuberculosis transmission and stigma may discourage clinic attendance and medication adherence. Behavioural factors, including social support and health literacy, influence long-term engagement in care [6]. Sputum conversion at 2 and 6 months is an objective indicator of treatment response and adherence. Delayed conversion is associated with poor compliance and increased transmission risk [7]. Quantitative assessment of knowledge, attitudes and practices combined with qualitative exploration of barriers can identify modifiable determinants of adherence [8]. Despite established treatment protocols, gaps in patient awareness persist in many high-burden settings. Identifying behavioural and educational deficits is critical for improving outcomes among HIV-positive individuals. Therefore, it is of interest to evaluate awareness, compliance and KAP related to tuberculosis treatment in HIV-positive patients and examine their association with sputum conversion outcomes.

## Materials and Methods:

A mixed-method design was utilized in this study, in which a questionnaire method was used at the TB-HIV clinic of a tertiary hospital for a period of six months. The study enrolled 120 patients aged 18-65 years, who were newly diagnosed with pulmonary tuberculosis, were HIV-positive and were started on anti-tuberculosis treatment using the RNTCP DOTS strategy after obtaining consent. Patients with extrapulmonary tuberculosis, drug resistance tuberculosis and with significant comorbidity were excluded. A carefully designed structured questionnaire was developed to measure the participant's knowledge, attitude and practice (KAP). KAP assessed comprehension of symptoms, treatment regimens, compliance, side effects and lifestyle modifications. Knowledge scored 0-10, attitude scored 0-8 and practice scored 0-7; higher scores reflecting higher knowledge levels and fidelity. Each correct/positive response scored 1 and each incorrect/negative response scored 0. Follow-up interviews were conducted to measure barriers to adherence and knowledge of TB using semi-structured qualitative questions. Sputum samples were collected at baseline and at 2 months and at 6 months for analysis of conversion rates. Data analysis was performed on SPSS version 26.0. Descriptive statistics were used to summarize demographic, clinical and KAP variables. Pearson correlation analysis and Chi-square tests were used to analyze the relationship between KAP scores and sputum conversion outcomes. A threshold of p-value <0.05 for statistical significance was used. Qualitative data were coded and thematically analyzed to support and complement the survey findings or results. The study was designed in a way to utilize triangulation of quantitative KAP assessment, clinical sputum results and qualitative data to highlight modifiable factors to improve adherence. Ethical clearance was acquired from the institutional review board and confidentiality, or an individual's right to privacy, was emphasized in the study.

## Results:

Out of a total of 120 study participants, there were 68 males and 52 females, with a mean age of 39.5 ± 10.8 years. The mean duration of HIV diagnosis was 5.2 ± 3.1 years. Baseline sputum positivity was established in each of study participants. The mean total KAP score was 17.3 ± 3.8, indicating moderate awareness. Good knowledge was noted in 40% of study participants, positive attitude in 47% and optimal practices were demonstrated in 45% of participants. At 2 months, 78% of study participants have converted their sputum and at 6 months, 92%. Higher KAP scores were significantly associated with prompt sputum conversion (p < 0.05). Table 1 (see PDF) describes demographic and clinical data for 120 HIV-positive participants. Most of them were males (57%), with a mean age of 39.5 years and an average duration of HIV infection of 5.2 years. All the patients were sputum positive at baseline, thus confirming active TB infection. Table 2 (see PDF) describes the knowledge of participants regarding TB treatment. The proportion of those who possessed good knowledge was only 40%, which means that the majority of the patients do not have adequate understanding of TB therapy and requirements for adherence. Table 3 (see PDF) reflects participants' attitudes toward TB management. Less than half of the respondents had favorable attitudes, while 20% had negative attitudes, due to hindrances that included stigma, fear of side effects and misunderstanding. Table 4 (see PDF) summarizes the respondents' self-reported practices regarding TB treatment. Only 45% adhered to whole DOTS, 50% remained in consistent follow-up and 42% checked for side effects, indicating a disparity between knowledge, attitude and practice. Table 5 (see PDF) demonstrates that at both 2 and 6 months, the sputum conversion rate increases as the knowledge score increases, suggesting that when a patient has a greater aptitude the treatment is more efficacious. Table 6 (see PDF) also depicts those individuals with favorable attitudes regarding TB had the highest reported sputum conversion outcomes; this again reflects that psychosocial factors are relevant to adherence. Table 7 (see PDF) depicts the participants who had the best practices; there is a better sputum conversion rate of these participants and that TB treatment adherence and even treatment behavior/engagement may substantially impact clinical outcomes. Table 8 (see PDF) suggests that total KAP score is positively correlated to sputum conversion rates at both 2 and 6 months, suggesting that overall increased awareness, positive attitudes and good practices contributed to better TB treatment outcomes.

## Discussion:

This mixed-method study evaluated knowledge, attitudes and practices related to tuberculosis treatment among HIV-positive individuals and examined their association with sputum conversion. The findings demonstrate moderate overall awareness but substantial behavioural gaps. Higher KAP scores were significantly associated with earlier sputum conversion at both 2 and 6 months. The 2-month conversion rate of 78% and 6-month rate of 92% indicate generally favorable outcomes. However, conversion was consistently higher among participants with good knowledge, positive attitudes and optimal practices [9]. Patients with poor knowledge demonstrated lower conversion rates. This suggests that awareness and adherence behaviours directly influence treatment response [10]. Knowledge deficits remain evident. Only 40% demonstrated good knowledge of tuberculosis therapy. Many patients lacked complete understanding of treatment duration and potential side effects. Inadequate knowledge may contribute to missed doses or premature discontinuation. Education is therefore essential for sustaining adherence in co-infected populations [11]. Attitudinal barriers also influenced outcomes. Less than half of participants demonstrated positive attitudes toward treatment. Negative attitudes were associated with delayed sputum conversion. Stigma, fear of adverse effects and pill burden likely contribute to poor engagement. Addressing psychosocial determinants is therefore critical [12]. The practice domain showed similar trends. Only 45% reported optimal adherence to DOTS. Participants with optimal practices achieved the highest conversion rates. This reinforces that behaviour, rather than knowledge alone, determines clinical outcomes. Structured reinforcement strategies are required to translate knowledge into sustained practice [13, 14]. The positive correlation between total KAP scores and sputum conversion supports the behavioural model of adherence. Patients with higher awareness and stronger engagement demonstrated better microbiological outcomes. These findings align with contemporary evidence showing that patient-centred education improves tuberculosis treatment success [15]. The mixed-method design strengthens interpretation. Quantitative correlations provide statistical evidence, while qualitative insights identify practical barriers such as travel burden, stigma and counselling gaps. Integrating behavioural assessment with objective sputum monitoring provides a comprehensive evaluation framework [16]. This study adds contemporary evidence from an integrated HIV-TB clinic setting. It quantifies the association between KAP domains and objectively measured sputum conversion. Few studies have combined behavioural assessment with microbiological outcomes in co-infected populations. The findings highlight modifiable behavioural determinants that may improve programme performance. Limitations include single-centre design and reliance on self-reported practices. Social desirability bias may influence responses. However, objective sputum outcomes enhance reliability of conclusions. Overall, tuberculosis control in HIV-positive individuals requires more than pharmacological therapy. Structured counselling, stigma reduction, adherence monitoring and patient education must be integrated into routine care. Closing the knowledge-attitude-practice gap is essential for sustaining sputum conversion and reducing transmission.

## Conclusion:

Data shows that improving knowledge, increasing positive attitudes and encouraging best practice, are strongly associated with increased sputum conversion rates in HIV-positive patients with TB. Patient education and structured counselling and adherence support are promising ways to close the knowledge-attitude-practice gap. Incorporating KAP assessments in routine HIV-TB care may also support adherence and ultimately improve outcomes.

## References

[1] Bosch G (2025). Enferm Infecc Microbiol Clin (Engl Ed)..

[2] Nacher M (2020). BMC Res Notes..

[3] Blanc FX (2020). N Engl J Med..

[4] Naidoo A (2022). BMJ Open..

[5] Vecchione MB (2021). Tuberculosis (Edinb)..

[6] Safe IP (2021). Front Immunol..

[7] Asemahagn MA (2021). BMC Pulm Med..

[8] Izudi J (2020). J Clin Tuberc Other Mycobact Dis..

[9] Bhatti Z (2021). East Mediterr Health J..

[10] Mulindwa F (2021). BMC Infect Dis..

[11] Saghazadeh A, Rezaei N (2022). Int Immunopharmacol..

[12] Hosu MC (2025). Pathogens..

[13] Patel A (2024). Cureus..

[14] Adisa R (2021). BMC Public Health..

[15] Woldesemayat EM, Gari T (2024). BMC Infect Dis..

[16] Abu-Humaidan AHA (2022). Front Public Health..

